# Impedance Flow Cytometry: A Novel Technique in Pollen Analysis

**DOI:** 10.1371/journal.pone.0165531

**Published:** 2016-11-10

**Authors:** Iris Heidmann, Grit Schade-Kampmann, Joep Lambalk, Marcel Ottiger, Marco Di Berardino

**Affiliations:** 1 Enza Zaden, Research and Development B.V. P.O. Box 7, 1600AA Enkhuizen, The Netherlands; 2 Amphasys AG, Technopark Lucerne, 6039 Root D4, Switzerland; Institute of Botany Chinese Academy of Sciences, CHINA

## Abstract

**Introduction:**

An efficient and reliable method to estimate plant cell viability, especially of pollen, is important for plant breeding research and plant production processes. Pollen quality is determined by classical methods, like staining techniques or *in vitro* pollen germination, each having disadvantages with respect to reliability, analysis speed, and species dependency. Analysing single cells based on their dielectric properties by impedance flow cytometry (IFC) has developed into a common method for cellular characterisation in microbiology and medicine during the last decade. The aim of this study is to demonstrate the potential of IFC in plant cell analysis with the focus on pollen.

**Method:**

Developing and mature pollen grains were analysed during their passage through a microfluidic chip to which radio frequencies of 0.5 to 12 MHz were applied. The acquired data provided information about the developmental stage, viability, and germination capacity. The biological relevance of the acquired IFC data was confirmed by classical staining methods, inactivation controls, as well as pollen germination assays.

**Results:**

Different stages of developing pollen, dead, viable and germinating pollen populations could be detected and quantified by IFC. Pollen viability analysis by classical FDA staining showed a high correlation with IFC data. In parallel, pollen with active germination potential could be discriminated from the dead and the viable but non-germinating population.

**Conclusion:**

The presented data demonstrate that IFC is an efficient, label-free, reliable and non-destructive technique to analyse pollen quality in a species-independent manner.

## Introduction

Since the frog-leg experiments of Galvani (1737–1798) and studies in Venus fly-trap by Burdon-Sanderson (1828–1905) numerous invasive and non-invasive techniques to analyse electric signalling across animal and plant tissues/organs have been developed [1,2]. Non-invasive, label-free dielectrophoresis (DEP) techniques take advantage of the reaction of dipolar particles to an applied, non-uniform electric field which allows the characterization of cells based on their conductive and permittive properties [3].

Biological membranes are semi-permeable bilayers of lipids and proteins encapsulating the cellular content of organelles, nucleus and cytoskeleton embedded in a conductive cytoplasm. An intact cellular membrane is not highly conductive and acts electrically as a combination of capacitor and resistor. The capacitance depends on the morphology of the membrane. As higher the morphological complexity, as higher the capacitance and as longer it takes before the membrane is charged. The analysis of different characteristics of single cells depends on the electrical interaction between the cellular surface and the surrounding medium and the permittivity of the cellular content. Therefore, the analysis of surface characteristics like cell size and membrane integrity does not require a high permittivity while the detection of cytoplasmic changes does [4]. Thinner membranes are quickly charged. Damaged membranes leak ions and become conductive which can be detected by specific electrodes. Typically, cell size, shape, and membrane properties are analysed between 10 kHz and 5 MHz, cytoplasmic analyses require frequencies above 10 MHz [4] characterized by the recorded amplitude and phase angle (impedance) at multiple frequencies by an impedance meter [5].

The analysis of electric properties in single cells by impedance flow cytometry (IFC) has been described in microorganisms to estimate viability, membrane potential, as well as cell size [6,7], in mammalian cells to characterize culture conditions and apoptosis [8] and to screen for methods to kill breast cancer cells [9]. The IFC system is based on the Coulter system [10] but uses a microfluidic chip which permits measurements in the radio frequency range from 0.1 to 30 MHz with alternating current (AC). An appropriate parameter to discriminate dead from viable cells proved to be the change of the phase angle of the detected impedance signal, while the amplitude provided information about cell size both displayed in a phase-amplitude dot plot. Depending on the chosen frequency, data of cell size, membrane capacitance, cytoplasmic conductivity of single cells, and cell concentration [7,11] are simultaneously obtained.

Viable and functional pollen grains are essential for sexual plant reproduction, the maintenance of genetic diversity, during breeding processes, and commercial seed production [12]. Developing pollen at a specific microspores stage are the base of numerous *in vitro* methods producing homozygous parental lines [13]. Mature pollen grains that are not directly used for pollination can be conserved by specific methods and stored for off-season crossing or germplasm preservation programs [14].

The characteristic steps of pollen development, pollen germination, and pollen tube formation have been intensively reviewed [15,16]. In brief, the developmental steps are accompanied by changes in cell size, composition of the cytoplasm, cell wall formation, nuclear divisions, and metabolic activity, but seem to occur not necessarily in a highly synchronised manner [17–19]. The carbohydrate type and content is species dependent [20], varies during the development [21], is affected by stress [22,23] and storage conditions [24] which further affects the membrane protective function of sucrose [25] with consequences for pollen viability and later germination capacity.

At dispersal from the anther mature pollen grains are released with a species-specific water content, which classifies pollen as partially hydrated (> 30% water content) or partially dehydrated (< 30% water content) [26].

Rehydration, the first step of the pollen towards germination [27], occurs within 5–60 minutes after contact to the stigma [26]. During this phase the metabolic activity of the pollen grain increases, which is essential for the later pollen tube formation [28]. Pollen tube elongation is controlled by active vesicle and organelle trafficking along an intact cytoskeleton [29]. Interferences, through genetic, environmental or biochemical factors, at any of the developmental steps can lead to aborted, small, shrunken, non-viable or non-germinating pollen [19,27,29,30] with subsequent effects on reproduction [31–35].

The quality of developing and mature pollen is determined by various classical, non-standardized methods, like staining with fluorescent/non-fluorescent dyes [36,37], size measurements [38] or pollen germination. However, each of these methods has its disadvantage with respect to general application, reliability, analysis time, labour intensity, genotype and species dependency in relation to pollen germination [39,40]. Until now, a general method that allows the simultaneous determination of pollen viability and prediction of germination has not been described.

Considering pollen as highly active hydrodynamic units [26,41] that change electric properties of their membranes and cytoplasm as they develop, mature and germinate, IFC should allow a quick and reliable characterisation of these plant cells. The knowledge of the specific hydrodynamic and metabolomic processes of pollen grains and their quality control plays an important role during pollen production, the development of pollen storage protocols, and of cause pollination.

Here we describe for the first time the use of impedance flow cytometry to analyse pollen development and viability in a high-throughput and species independent manner, which also predicts pollen germination.

## Material and Methods

### Sample preparations

Pollen donor plants (standard lines and breeding material, Enza Zaden, The Netherlands) were grown in heated greenhouses under standard conditions. Developing pollen from microspore (prior pollen mitosis I) to immature stage (after pollen mitosis I) of tobacco (*Nicotiana tabacum*, SR1) or mature pollen (after pollen mitosis II [42]) of cucumber (*Cucumis sativus*) were isolated by gentle squeezing excised anthers directly in IFC measurement buffer (tobacco AF4, cucumber AF5, Amphasys, Switzerland). Dry, mature pollen (pepper (*Capsicum annuum*), tomato (*Solanum lycopersicum*) 'Moneyberg', 'Moneymaker', beef and cherry types) were harvested by shaking open flowers inserted into Eppendorf tubes with an electric tooth brush. The collected pollen were resuspended in IFC buffer (tomato, pepper AF6, Amphasys) and filtrated through 50 μm (tobacco, tomato, pepper) or 150 μm (cucumber) filter units (CellTrics®, Sysmex Partec, Germany) prior analysis. The liquid impedance of the IFC measurement buffers was adjusted to10-30 kΩ.

Viability and developmental stage were measured at 0.5 and/or 12 MHz at the default settings (Trigger level 0.1 V, Modulation 3, Amplifier 6, Demodulation 2, Pump 60 rpm) by loading the pollen samples onto a 120 μm (tobacco, tomato, pepper) or 250 μm (cucumber) channel chip, inserted in an impedance flow cytometer type AmphaZ30 (Amphasys, Switzerland). Data of at least 5 x 10^3^ cells per sample with a concentration between 5 x 10^4^ and 10^5^ cells/ml were collected and analysed using AmphaSoft v1.2 and converted to the 2.0 version for a better resolution of the plots (Amphasys, Switzerland).

To determine the optimal conditions to create non-viable controls that allow a clear discrimination between viable and non-viable cells, 100 μl aliquots of pollen suspension in closed 250 μl PCR vessels (Greiner) were exposed to a temperature gradient ranging from 40–60°C for 15 minutes (T100 Thermo cycler, Bio-Rad). Aliquots were cooled down to room temperature and filled up to 1 ml with the same IFC buffer before analysis.).

For cell size determination polystyrene beads of 20, 25 and 30 μm (Sigma) were added in a 1:200 dilution to the filtrated pollen samples.

### Classical viability and stage determination

To compare the IFC data with a classical method pollen viability was determined in parallel to IFC by classical fluorescein diacetate (FDA; Sigma 50 μg/ml in DMSO) staining. For the analysis of the developmental stage microspores from five to six buds were isolated in IFC buffer, precipitated after IFC measurements, stained with 4’,6-diamidino-2-phenylindole (DAPI; Sigma [43]), and observed under a fluorescent microscope (Olympus IX70). Images were taken with a DP70 camera using CellSens software (Olympus).

### Detection of starch accumulation

Pollen development is accompanied by starch accumulation. For starch detection excised anthers of tobacco flowers were squeezed in Lugol’s potassium iodine solution (Sigma [44]) and directly imaged.

### Pollen germination

Pollen germination was determined on the same sample as used for the IFC analysis. Pollen grains were either recollected after the IFC measurement or the remaining aliquot which was not used for IFC was prepared for the germination assay. Samples were centrifuged at 4000 rpm for five minutes, the IFC buffer was replaced by 200 μl germination buffer (tomato and pepper, Brewbaker and Kwak 1963 [45]; cucumber, Vižintin and Bohanec 2004 [46]), spread into a 125 μl Gene frame (AB-0578, Life Technologies) mounted on a standard glass slide, and incubated for 30 minutes (cucumber) or two (tomato, pepper) hours in closed *in vitro* vent boxes (Duchefa, The Netherlands) lined with wet filter paper (Whatman) at room temperature. The germination data were acquired by analysing at least 200 pollen grains per sample. Inactivation experiments were performed as described above but with a temperature gradient ranging from 20–40°C.

The experiments concerning viability analysis and germination inactivation were performed with three biological replicates collected at different days over a period of two months, the germination prediction was performed with three individual tomato lines collected on the same day.

## Results

### IFC detects different developmental stages

Immature pollen grains (microspores) are essential for the *in vitro* production of homozygous parental lines via the androgenetic pathway. In many doubled haploid protocols the stage between uni-nucleate and early bi-nucleate are the most responsive ones [47]. Their correct stage and concentration of viable cells at the start of the culture is predominantly analysed manually by DAPI and FDA staining, respectively.

**Fig 1:**
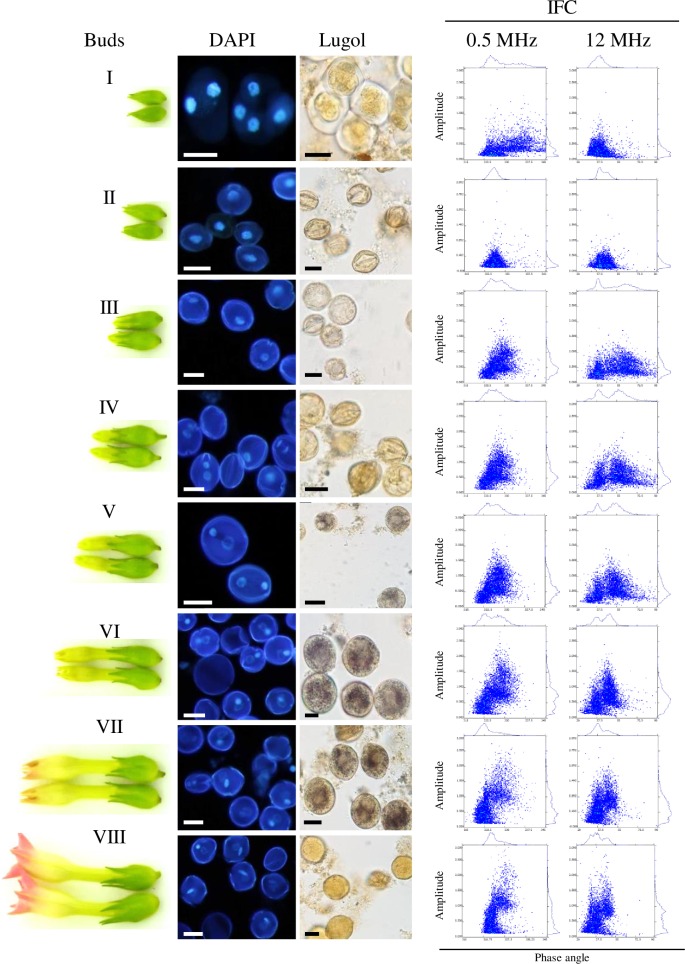
Analysis of developing tobacco pollen. Developmental stages of tobacco pollen according to flower bud size (Buds) was followed by stage determination (DAPI), starch accumulation (Lugol) and IFC analysis at 0.5 and 12 MHz. The stages (I-VIII) represent I, tetrad; II, uni-nucleate, III, late uni-nucleate; VI–VII, binucleate, VIII, mature pollen grains. Scale bars 20 μm

To demonstrate that developmental differences can be detected by IFC we isolated microspores from different bud sizes of tobacco and analysed them by nuclear staining (DAPI), potassium iodine (Lugol) staining for starch detection, and IFC at 0.5 and 12 MHz (Fig 1). Along with the increasing tobacco flower size (Fig 1 Buds I—VIII) the progression of pollen development from tetrad to late binucleate stage (Fig 1 DAPI) and starch accumulation (Fig 1 Lugol) between stage V and VII was detected. The Amphasoft dot plots of the analysed stages showed two clearly distinguishable populations throughout the tested stages (Fig 1 IFC). While the tetrad stage (Fig 1 Buds I, DAPI) analysed at 0.5 MHz (Fig 1 IFC) show two distinguishable population that vary in size, displayed by the amplitude (y-axis) and phase angle (x-axis), the analysis at 12 MHz shows only one population with strong variation in size and cytoplasmic impedance which is probably a mix of tetrads and release young microspores. Uni-nucleate microspores showed one population at 0.5 MHz but two with a different phase angle when analysed at 12 MHz (Fig 1 Buds II, IFC). All later stages (III–VIII) analysed at 0.5 MHz showed one population with a lower phase angle that remained at the same position throughout the measurements while the second, with the higher phase angle and a shift to the right, definitely changed in both amplitude and phase value (Fig 1 IFC). At the stage III and IV the population with the high phase angle analysed at 12 MHz showed a broad variation from 40° to 90° (Fig 1 IFC) which was narrowed at the point of first starch detection at stage V (Fig 1 Lugol and IFC) and subsequent later stages to a phase angle value not higher than 55° (Fig 1 IFC). The comparison of the IFC data with the DAPI staining strongly suggests that the population with the higher and changing phase angle corresponds to developing microspores as it is expected that this group of microspores differs in size and cytoplasmic activity from dead microspores that did not display a change in the phase angle.

### IFC discriminates efficiently between viable and non-viable pollen populations

**Fig 2:**
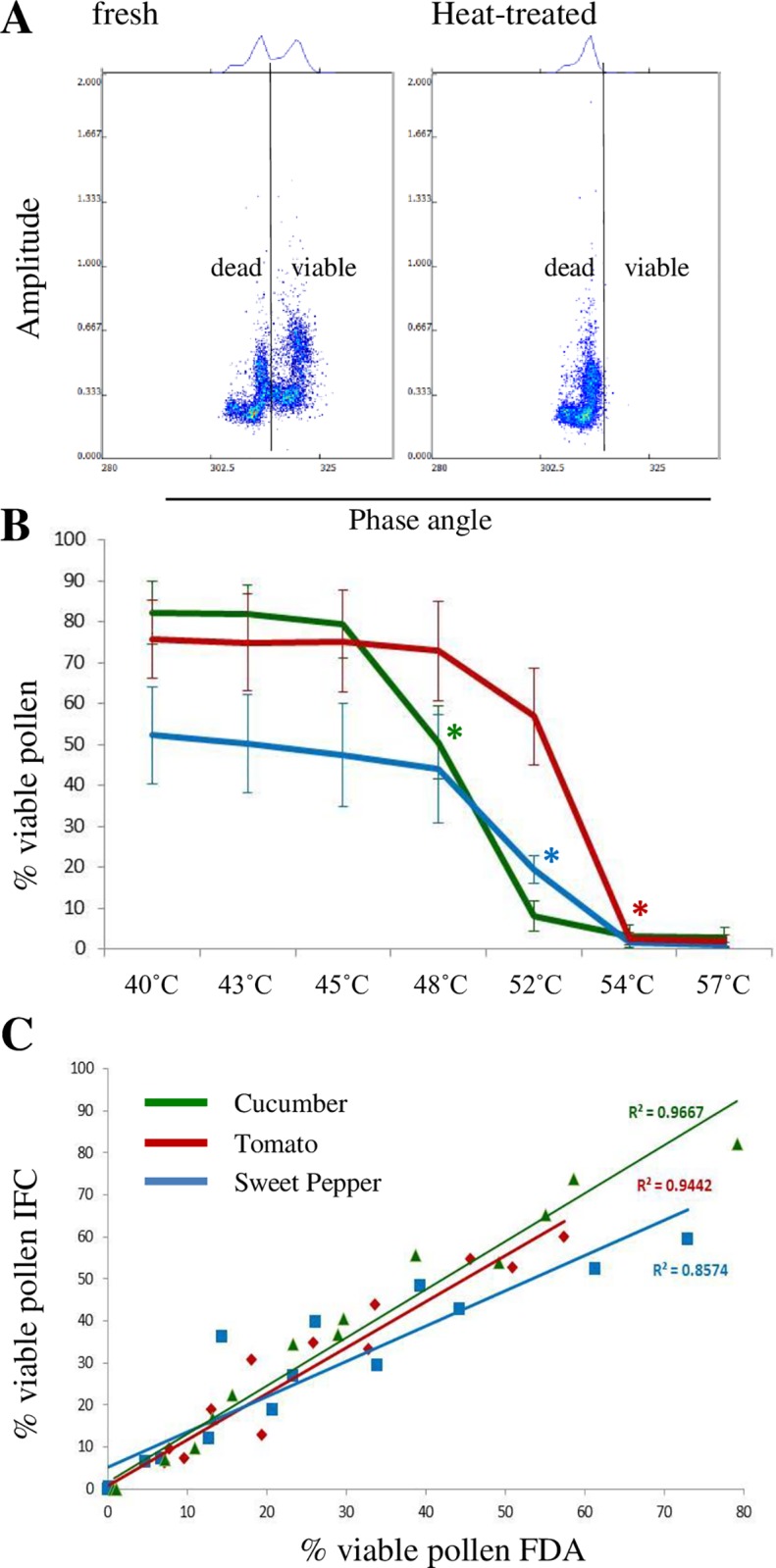
IFC discriminates between viable and non-viable pollen populations. Pollen of three different species, cucumber (green), sweet pepper (blue) and tomato (red) were inactivated by heat. The effect of the heat treatment on pollen viability was analysed by IFC and classical FDA staining. **A,** AmphaSoft dot plots and histograms at 0.5 MHz of fresh (left) and heat-treated (right) tomato pollen populations; **B,** Temperature-dependent decrease of pollen viability detected by IFC at 12 MHz, * p = < 0.05; **C,** Correlation of IFC data at 12 MHz with classical FDA staining.

To identify the previously observed populations (Fig 1) we compared freshly harvested, mature pollen of various species with heat-treated aliquots. We found that all untreated pollen samples showed two clearly distinguishable populations with low and high phase angles (Fig 2A, left) while heat-treated pollen showed only one with a low phase angle (Fig 2A, right) which identified the population with the higher phase angle of Fig 2A as the viable one because it is no longer present after heat-inactivation. This pattern was confirmed in all other tested species treated in the same way, in naturally aged pollen e.g. in pollen harvested from old flowers (cucumber four days post anthesis), or pollen (tomato) stored for more than seven days at room temperature. Alternatively, a non-viable pollen population can be determined using polystyrene beads which indicate a certain size but should not show a variation in phase angle. To demonstrate the suitability of beads, we first analysed beads of 20, 25, 30 μm and a mix thereof with a 120 μm chip at 0.5 and 12 MHz (S1A–S1D Fig). As expected, the beads could be distinguished according to their size and displayed no change in the phase angle. The bead mix (S1D Fig) was then added to fresh and heat-treated tomato pollen samples (S1E–S1G Fig) and analysed in the same way. As previously seen in the tobacco samples the fresh sample showed two populations with a different phase angle and variation in size (S1D Fig). At 0.5 MHz the left population was almost indistinguishable from the beads, while the population with the high phase angle corresponded to the bead size of 30 μm (S1F Fig). At 12 MHz the beads showed a very different phase angle than the two pollen populations (S1F Fig). In the heat-treated sample (S1G Fig) the with the higher phase angle was absent at both, 0.5 and 12 MHz indicating that this particular population must have been the viable one. The intensified signal of the heat-treated pollen population between the signal of 25 and 30 μm beads at 0.5 MHz (S1G Fig) showed that the heat-treated pollen shifted in both phase angle and size.

The gating function of the software allows the automatic calculation of the ratio of dead/viable fractions (line gating) or individual populations (polygon gating) which was not used in the presented images to maintain a clear figure, instead the separation between dead and viable is indicated by a line, the beads are marked in red (S1E–S1G Fig).

The data demonstrate that dead pollen are not only smaller than viable ones but also behave almost the same way as polystyrene beads in the applied AC field. The observed “J”-shape of the bead and tomato populations at 0.5 MHz (S1A–S1D Fig) is due to the natural size/shape variation of the beads and their individual position within the channel. As the flow rate in the squared microfluidic channel was around 0.5 ml/min (corresponds to a Reynolds number of around 70), the cells are likely aligned along four streams, an effect also known as inertial focussing [48]. In this fluidic situation and within the used electrode configuration the system detects two preferred signal amplitudes, a smaller amplitude for the cells in the middle of the channel, and a larger amplitude for cells on the top or bottom of the channel, which are closer to the electrodes. The slight phase shift leading to the J-shape is an effect of electronic cross-talk between the electrode pairs [49]. This cross-talk is substantial at low frequencies (0.5 MHz), but almost inexistent at high frequencies (12 MHz). During the estimation of the optimal inactivation temperature we found that pollen of different species reacted differently to the applied temperature. The viability of cucumber pollen significantly declined after a 15 minute heat treatment at 48°C while the viability of sweet pepper and tomato pollen declined at higher temperatures. Above 54°C none of the tested pollen was viable anymore (Fig 2B).

We compared our IFC data with classical FDA staining by mixing fresh pollen with heat-treated (60°C) ones in fixed ratio reducing the amount of viable pollen from 100% in steps of 25% down to zero. For the three analysed species, cucumber, sweet pepper, and tomato a high correlation (R^2^ = 0.85–0.96) between FDA and IFC (Fig 2C) was found.

### IFC predicts pollen germination in tomato

In parallel to the previously described heat-treatments at 40–60°C we performed pollen germination assays but no pollen germination was observed in this temperature range despite the detected viability (Fig 2B). Therefore, we chose a lower temperature range (20–40°C) to find the critical conditions that would inactivate germination.

When comparing pollen germination of samples before and after measurements we found that pollen germination is not affected by the passage through Ampha Z30. Since the re-collection required more handling we continued the pollen germination assay on the remaining aliquot which was not used for the measurement.

**Fig 3:**
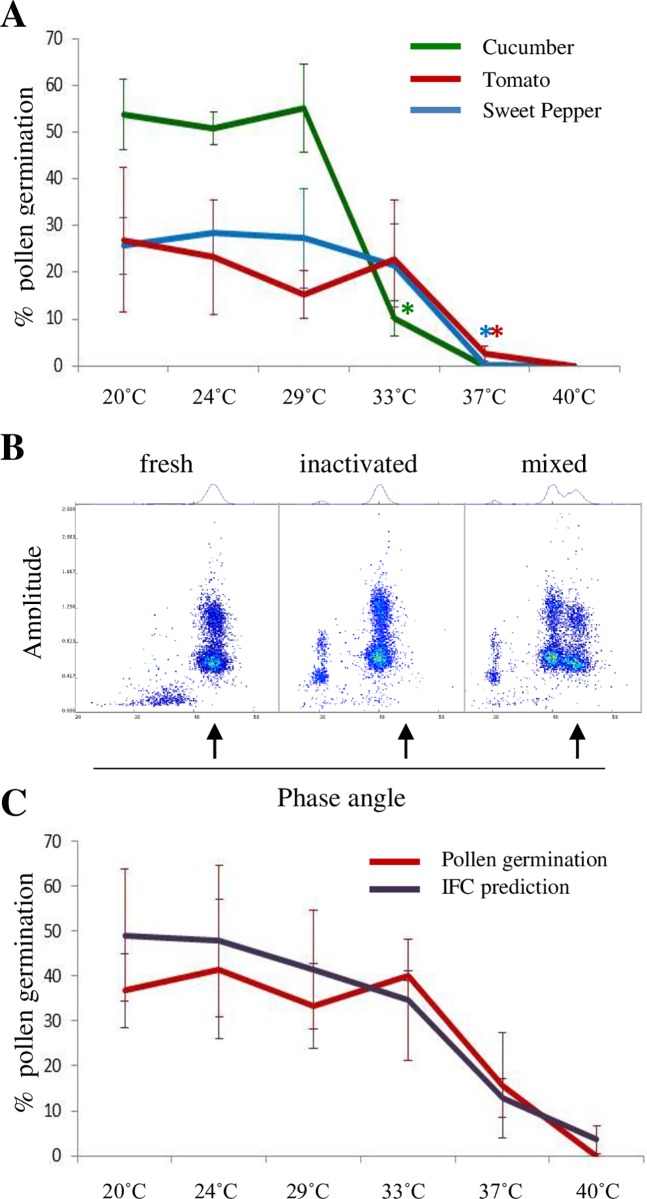
Heat inactivation of pollen germination and prediction by IFC. **A,** Germination of fresh and heat-inactivated pollen samples, cucumber (green), sweet pepper (blue), and tomato (red), * p = < 0.05; **B,** AmphaSoft dot plots and histograms of fresh (left), 40°C-inactivated (middle), and a mixed pollen population. The arrow points at the fresh and germinating population **C,** IFC prediction of tomato pollen germination (purple) and *in vitro* pollen germination (red) after heat-inactivation;

As seen in the earlier experiments, cucumber pollen was more sensitive to a 15 minutes heat treatment than sweet pepper or tomato pollen. Cucumber pollen germination was significantly inhibited at a lower temperature (33°C) than in sweet pepper and tomato (37°C). It was further noticed that with increasing temperatures, above 33°C, pollen tubes shortened and beyond 37°C no pollen germination could be observed (Fig 3A).

Tomato pollen can be harvested in a high quantity from a few plants. Therefore, we focussed on tomato to find IFC settings that would predict pollen germination. Pollen samples were collected and treated as described before. To identify the population with active germination capacity we mixed a fresh pollen sample with aliquots of 40°C-inactivated ones of the same genetic background in defined ratios, pre-checked the instrument settings (different MHz) for an optimal resolutions. The previously used frequency of 0.5 MHz was not suitable for the discrimination purposes, so we chose 3 and 12 MHz, the frequencies expected to indicate membrane and cytoplasmic integrity [4], with an adaptation of the settings (Trigger 0.05 V, Modulation 4, Amplifier 6, Demodulation 1). We found that the population with the highest phase angle was missing in the inactivated sample at 3 and 12 MHz and therefore must have been the pollen population with germination capacity. Because of a clearer separation of the inactive and active populations, and minimized undesired electronic cross talk at 12 MHz we used this frequency as standard to predict pollen germination (Fig 3B and S2A–S2E Fig). We confirmed our findings by exposing freshly harvested pollen of different tomato lines to a temperature gradient (20–40°C) and found no significant difference between the IFC data predicting pollen germination and *in vitro* germination (Fig 3C and S2F–S2J Fig) when a germination-inactivated control was used as a negative reference.

The high variation per data point is due to the fact that different genotypes were used and that the experiments had to be repeated over several days. It is likely that the greenhouse conditions per day have varied in terms of light intensity and relative humidity, which might have had affected the pollen quality.

## Discussion

IFC has been successfully used for cellular characterisation in bacterial and mammalian systems [6,8,9]. Analysing plant cells and in particular the quality of pollen in an efficient and reliable manner is not only of interest of plant breeding and seed production industries, but plays also a role in scientific projects investigating pollen development [30,50], pollen-related heat or cold tolerance [22,23], male-sterility systems [51], the establishment of pollen storage or doubled haploid protocols [13,14]. The most critical argument against all methods analysing pollen viability is the missing relevance for pollen germination [39]. Viability is a pre-requisite but no warranty for pollen germination, successful fertilisation and fruit set. This cascade of processes depends on external/internal stimuli and specific gene expression of both male and female partner [52–55]. The presented work focusses on viability determination of developing and mature pollen and the prediction of germination.

We have shown that different steps in pollen development can be visualized by IFC. Due to the non-synchronous pollen development and subsequent variations in size and cytoplasmic dynamics [19] the populations are not displayed as a sharp dot but rather as a cloud. The phase angle shift occurring during the binucleate stage (stage VI–VII) is likely caused by an enhanced carbohydrate synthesis and starch accumulation. At maturation stage (VII–VIII) the viable pollen were represented in a compacter cloud than in the stages before suggesting a higher cell synchronisation and a development arrest prior germination [25].

The different populations were identified by heat-treatment or natural aging either at the plant or by wrong storage conditions which caused a decrease in size and shift in phase due to the induced membrane cytoplasmic damages [25]. The discrimination between dead and viable cells can be detected at all frequencies and applied at any stage to all species.

We found that the cells are still functional after the measurement and the passage through the instrument which opens opportunities to expose cells to biotic or abiotic stresses, analyse their properties, and subject them directly to culture, molecular, biochemical characterization, or other purposes like storage or pollination.

The size based stage determination by IFC should also allow the detection of off-type pollen grains as described for the volume-based detection of aneuploid pollen grains [56]. In preliminary experiments we were able to discriminate between diploid and tetraploid tomato individuals of the same genetic background but because of the natural pollen size variation we could not clearly detect a contamination of diploid pollen within a haploid population.

The advantage of the presented method over classical viability methods involving dyes or fluorochromes, is that IFC allows a high throughput viability screening of e.g. large mutant populations, where 5 x 10^3^ cells, depending on the concentration, can be analysed in less than ten seconds in a standardisable manner.

The observation that the technique can discriminate between different stages and can also detect changes in the cytoplasm encouraged us to investigate if pollen germination can be predicted. The dielectric principles of IFC predict an optimal frequency range for cell size analysis between 0.01 and 20 MHz, membrane integrity between 1 and 10 MHz, and cytoplasmic conductivity above 10 MHz [2]. Pollen becomes metabolic active prior germination, thus changes in membrane integrity and cytoplasmic conductivity should be detectable when comparing germinating active with inactive pollen [28].

The finding that we could discriminate between pollen populations with and without germination capacity at 3 and 12 MHz demonstrates that we were able to detect the described metabolic changes pollen germination [19,26–28,41]. Pollen incapable of active re-hydration has a lower water content and likely a slower (passive) up-take than hydrated pollen and subsequently a different resistance when exposed to an electric field, which allows the discrimination between the two populations by IFC and leads to a high predictability of the IFC data for *in vitro* germination as only hydrated pollen is able to germinate [26,27,41]. While the separation of germinating from non-germinating and dead pollen is obvious in the mix of the two differently treated pollen samples (fresh/active versus germination inactivated, S2A–S2E Fig), the separation is less obvious in a natural population exposed to a temperature gradient (S2F–S2J Fig). The later can be explained by the expectation that not all pollen have developed synchronously [19]. Subsequently, they will not hydrate, be metabolic activated, and react to heat in a synchronized manner and therefore display a gradient from activated to completely inactivated. Thus, a germination–inactivated control is indispensable for this type of assay.

The effect on heat stress on pollen development and carbohydrate content in tomato was predominantly performed on whole plants [57,58]. Heat treatment applied to hydrated tobacco pollen but not submerged pollen affected the cytoskeleton and sucrose synthase deposition in pollen tubes during germination [29]. We chose to treat the pollen in liquid for an equal distribution of heat during the short-term treatment. The inhibition of tomato pollen germination between 37 and 40°C is in line with previous publications demonstrating that a treatment of 38°C negatively affects pollen germination in tobacco and tomato [29,59,60]. The ability of tomato pollen to germinate after long-term storage or heat stress depends on the biosynthesis of polyamines [60,61], or *β*-alanine [62] with a role for specific amino acid transporters during de-/hydration phases [63]. In tobacco (*Nicotiana tabacum*) pollen specific peptides are dephosphorylated during pollen activation [28].

The applied heat stress must have affected one or more described factors responsible for successful pollen germination. While gene expression or enzymatic activities cannot be analysed directly by IFC, consequences thereof can be observed if they affect the cellular integrity and the reaction to an electric field.

The applied combination of controlled and adjustable temperature gradient and IFC analysis opens opportunities to screen for genetic variation in temperature tolerance [22,23].

We have shown that IFC is a quick and reliable method to analyse pollen quality in a species-independent manner. To our knowledge, this is the first demonstration of an easily applicable and non-destructive method that allows the simultaneous discrimination between dead, viable and germinating pollen populations in a single measurement which opens opportunities to screen for any condition that affects pollen development and quality. Future technical IFC developments should allow the separation of the observed populations and in combination with molecular and biochemical methods support the future characterization of specific pollen populations.

## Supporting Information

S1 Fig Size determination of beads, viable and dead pollen of tomato. AmphaSoft dot plots and histograms at of polystyrene beads and pollen. **A,** 20 μm beads; **B**, 25 μm beads; **C,** 30 μm beads; **D**, equal mix of A-C; **E**, fresh tomato pollen; **F**, fresh tomato pollen supplemented with bead mix (D); **G**, inactivated tomato pollen supplemented with bead mix (D). The beads mix (D) has been marked red in the pollen samples (F and G) analysed at 0.5 MHz for a better visualisation, and marked with a red “**B”** at 12 MHz.
(TIF)

S2 Fig IFC analysis predicts pollen germination. AmphaSoft dot plots and histograms of germination active and inactivated tomato pollen populations analysed at 12 MHz. **A-E**, mixed ratios of active (**A**) and 40°C-inactivated pollen (**E**), the ratio are indicated in the plots (**B-D**); **F-J**, pollen population exposed to different temperatures as indicated in the plots. The line marks the convergence zone between viable and germinating pollen population, the position of the dead pollen population is marked with a red “**D**”.
(TIF)
