# Supplementary figures and images for: Impedance Flow Cytometry: A Novel Technique in Pollen Analysis
Source: PLoS One. 2016 Nov 10;11(11):e0165531. doi: 10.1371/journal.pone.0165531 (PMC5104384; doi:10.1371/journal.pone.0165531)

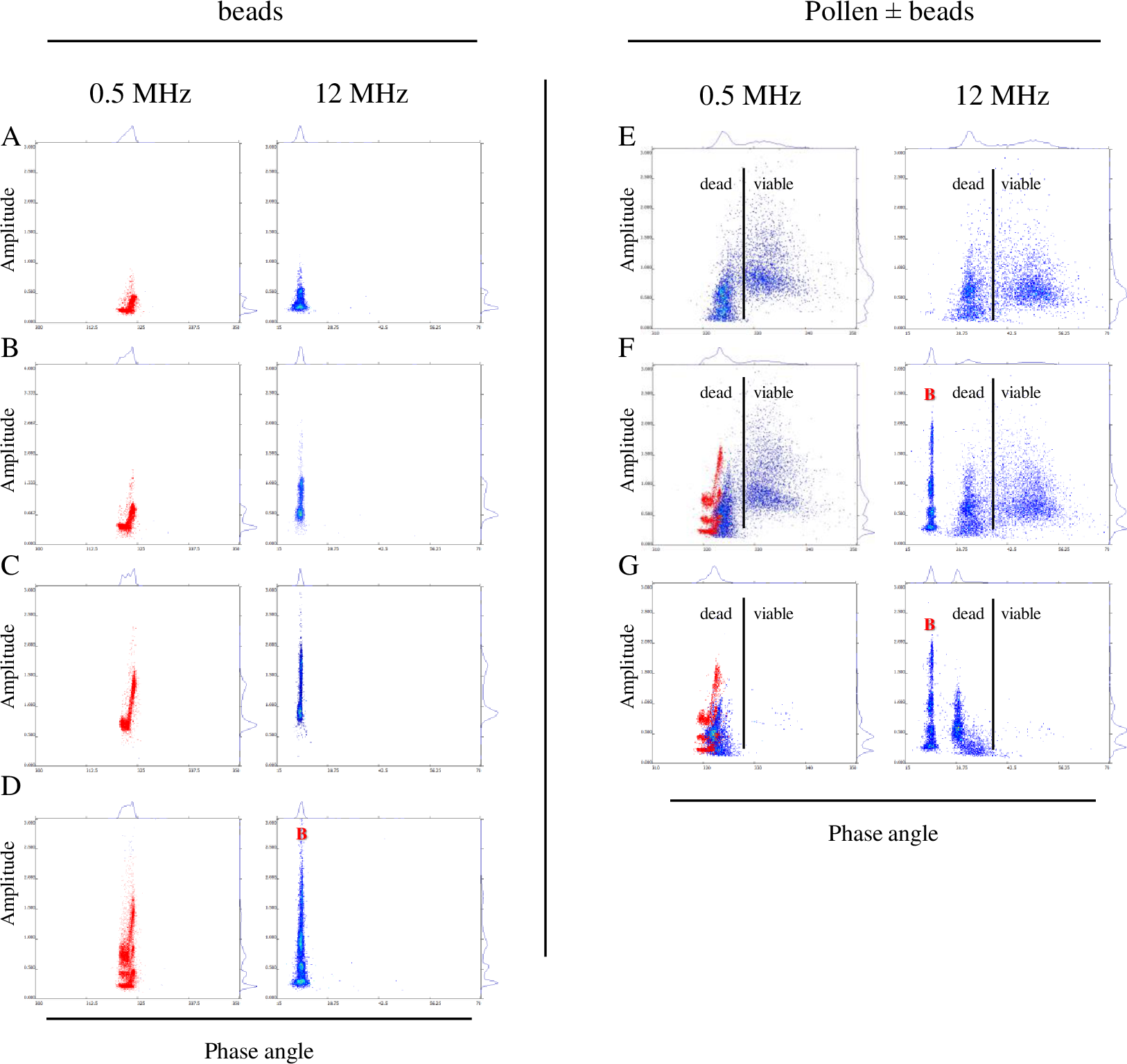

Supplement: S1 Fig — AmphaSoft dot plots and histograms at of polystyrene beads and pollen. A, 20 μm beads; B, 25 μm beads; C, 30 μm beads; D, equal mix of A-C; E, fresh tomato pollen; F, fresh tomato pollen supplemented with bead mix (D); G, inactivated tomato pollen supplemented with bead mix (D). The beads mix (D) has been marked red in the pollen samples (F and G) analysed at 0.5 MHz for a better visualisation, and marked with a red “B” at 12 MHz. (TIF) [file pone.0165531.s001.tif]

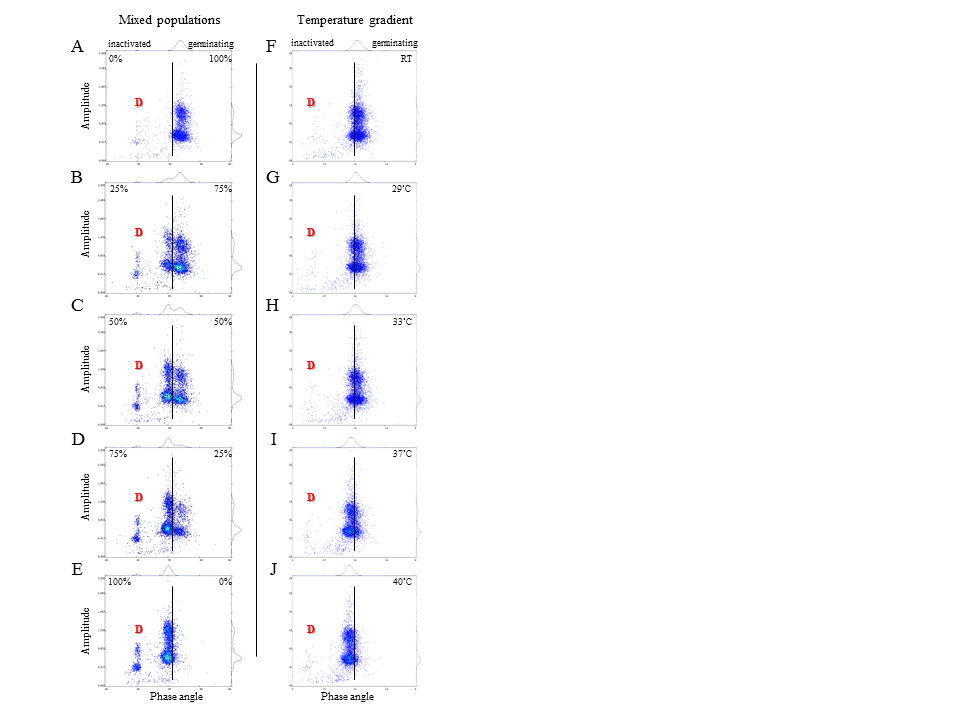

Supplement: S2 Fig — AmphaSoft dot plots and histograms of germination active and inactivated tomato pollen populations analysed at 12 MHz. A-E, mixed ratios of active (A) and 40°C-inactivated pollen (E), the ratio are indicated in the plots (B-D); F-J, pollen population exposed to different temperatures as indicated in the plots. The line marks the convergence zone between viable and germinating pollen population, the position of the dead pollen population is marked with a red “D”. (TIF) [file pone.0165531.s002.tif]
